# Obstructive Sleep Apnea: The Expanding Role of Dental Sleep Medicine—A Systematic Review of Mandibular Advancement Devices, Treatment Efficacy, and Occlusal Complications

**DOI:** 10.3390/dj14010062

**Published:** 2026-01-17

**Authors:** Jędrzej Szmyt, Tymoteusz Szczapa, Maksymilian Chyła, Adam Bęben, Izabela Maciejewska

**Affiliations:** Department of Prosthodontics, Medical University of Gdansk, Marii Skłodowskiej-Curie 3a, 80-210 Gdańsk, Poland; jedrek.szmyt@gumed.edu.pl (J.S.); maksymilian.chyla@gumed.edu.pl (M.C.); izabela.maciejewska@gumed.edu.pl (I.M.)

**Keywords:** obstructive sleep apnea (OSA), sleep-disordered breathing, mandibular advancement device (MAD), daytime sleepiness, oral appliance therapy, upper airway obstruction, polysomnography (PSG), apnea-hypopnea index (AHI)

## Abstract

**Background:** Obstructive sleep apnea is characterized by recurrent upper airway obstruction during sleep, leading to intermittent hypoxemia, sleep fragmentation, and excessive daytime sleepiness. Affecting up to 11% of the adult Polish population and more commonly diagnosed in men, OSA poses a major public health concern due to its association with cardiovascular, metabolic, and neurocognitive complications. This review summarizes the current evidence on diagnostic methods, risk factors, and therapeutic approaches, with particular emphasis on oral appliance therapy using mandibular advancement devices (MADs). **Methods:** A systematic literature review was conducted using the PubMed and Scopus databases, covering publications from 2020 to 2025, including clinical trials, meta-analyses, and systematic reviews evaluating the efficacy and safety of MAD therapy. **Results:** Findings demonstrate that MAD effectively reduces apnea–hypopnea index (AHI) values, improves oxygen saturation, and alleviates snoring and daytime fatigue, offering a patient-tolerable alternative for those intolerant to continuous positive airway pressure (CPAP). However, long-term use may cause occlusal or dental changes. Novel techniques, such as Er:YAG laser therapy, show potential in treating mild OSA. Moreover, epidemiological data suggest a correlation between tooth loss and an increased risk of OSA, particularly among men over 65. **Conclusions:** Dentists play a pivotal role in early detection, screening, and interdisciplinary management of OSA, underscoring the importance of collaboration between dental professionals and sleep medicine specialists for comprehensive care.

## 1. Introduction

Obstructive sleep apnea (OSA) is one of the most common sleep-related breathing disorders, characterized by recurrent episodes of partial or complete collapse of the upper airway during sleep. These events lead to intermittent hypoxemia, sleep fragmentation, and clinical symptoms such as loud snoring, excessive daytime sleepiness, and chronic fatigue. It is estimated that OSA affects between 3% and 11% of the adult Polish population, with a higher prevalence in men. Untreated OSA significantly increases the risk of cardiovascular disease, metabolic dysfunction, and even vehicle accidents, making it a major public health concern [1].

Continuous positive airway pressure (CPAP) remains the gold standard for OSA treatment, though long-term adherence and patient acceptance are often limited [1]. Consequently, alternative therapeutic strategies have been investigated, aiming to provide effective management of the disease in concert with patients’ greater comfort and tolerability. Among these alternatives, oral appliances, particularly mandibular advancement devices (MADs), have attracted increasing clinical relevance. MADs compel a forward reposition of the mandible, thereby enlarging the upper airway space, reducing pharyngeal collapse, and improving airflow during sleep. Numerous clinical studies and meta-analyses demonstrated MAD efficacy in reducing snoring and curing mild-to-moderate OSA [2]. Thus, MADs serve as an alternative treatment option for patients with severe OSA who are intolerant to CPAP therapy.

The purpose of this paper is to present current evidence regarding the indications, effectiveness, and potential side effects of oral appliance therapy in OSA, with particular emphasis on the role of dentists in the interdisciplinary management of this condition. The epidemiological studies showed the correlation between tooth loss and the occurrence of OSA [3]. It has been proven that the risk of OSA development correlates with the number of lost teeth, regardless of age and sex; however, this correlation is more pronounced in men and individuals over the age of 65. The risk of developing OSA increases by 25% with the loss of 5 to 8 teeth, 36% with the loss of 9 to 31 teeth, and 61% in cases of complete edentulism [2].

## 2. Materials and Methods

Obstructive sleep apnea (OSA) severity is primarily quantified by the apnea–hypopnea index (AHI), which reflects the number of breathing disruptions per hour of sleep. Effective treatment aims to substantially reduce AHI values, thereby decreasing the risk of associated cardiometabolic and neurocognitive complications.

**Figure 1:**
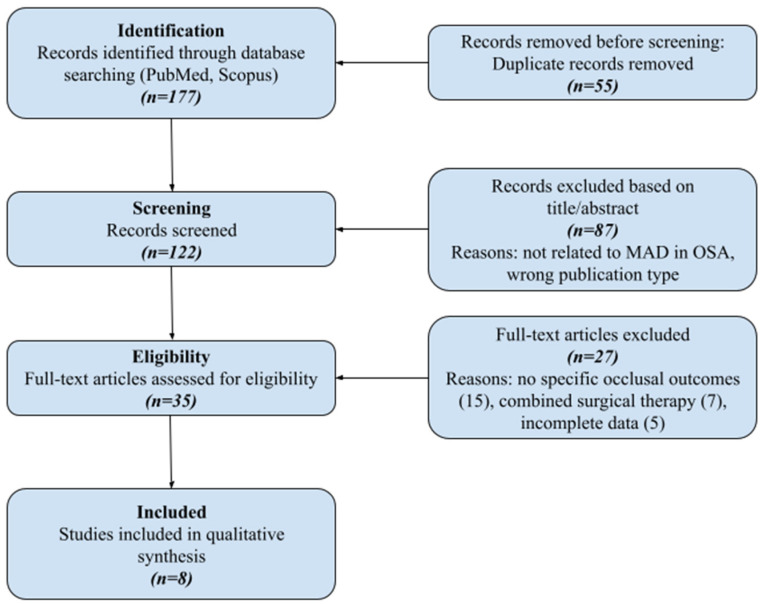
PRISMA flow diagram detailing the study selection process.

This study consists of two parts: the narrative review, which introduces the reader to the OSA problem, and a systematic review of the literature on obstructive sleep apnea (OSA) and the use of oral appliances, in particular mandibular advancement devices (MADs), in its treatment. The literature search was performed in the PubMed and Scopus databases, covering the period from January 2020 to September 2025. This systematic review was conducted in accordance with the Preferred Reporting Items for Systematic Reviews and Meta-Analyses (PRISMA) statement. A PRISMA flow chart has been constructed (Figure 1), along with a PRISMA 2020 Checklist (File S1). The following keywords and their Polish equivalents were used in various combinations: obstructive sleep apnea, mandibular advancement device, oral appliance therapy, dental sleep medicine, and bite changes in MAD therapy. Table 1 presents the list of the final included articles.

The analysis included clinical trials, randomized controlled trials, meta-analyses, systematic reviews, and case reports on the diagnosis, efficacy, and potential complications of MAD therapy in patients with OSA. The exclusion criteria were as follows: lack of access to a full text of a manuscript, language other than English or Polish, studies focusing exclusively on surgical or pharmacological interventions, and publications older than 5 years.

Literature searches were conducted in PubMed and Scopus using the following Boolean strategy, limited to Title/Abstract fields:

“(Patient Selection[Title/Abstract] AND Efficacious[Title/Abstract] AND Mandibular[Title/Abstract]) OR (Occlusal Side Effects[Title/Abstract] AND Mandibular Advancement[Title/Abstract]) OR (Treatment Outcomes[Title/Abstract] AND Mandibular Advancement[Title/Abstract] AND Mild[Title/Abstract]) OR (Definition[Title/Abstract] AND Effective[Title/Abstract] AND Oral Appliance[Title/Abstract] AND Snoring[Title/Abstract]) OR (irrespective[Title/Abstract] AND amount[Title/Abstract] AND movement[Title/Abstract] AND young[Title/Abstract]) OR (Diagnostic[Title/Abstract] AND Therapeutic[Title/Abstract] AND Indications[Title/Abstract] AND Mandibular[Title/Abstract]) OR (Impact[Title/Abstract] AND Occlusal Dynamics[Title/Abstract] AND Tooth Alignment[Title/Abstract]) OR (Dental side effects[Title/Abstract] AND 10-year[Title/Abstract]) OR (Obstructive sleep apnea AND Dental sleep medicine AND Therapy with sleep apnea devices)”.

The following aspects were evaluated:

- OSA diagnostic criteria, particularly questionnaires (STOP-BANG) and polysomnography (PSG);
- values of the apnea–hypopnea index (AHI) and respiratory disturbance index (RDI), used to determine the severity of the disease;
- patient populations eligible for MAD therapy,
- effectiveness of oral appliances in reducing the number of apneic events, improving oxygen saturation, and enhancing subjective sleep quality;
- potential adverse effects, including occlusal changes, temporomandibular joint symptoms, and the impact of therapy on dentition and periodontal tissues.

Data extracted from selected publications were compared and synthesized to assess the efficacy and safety of oral appliance therapy as an alternative or adjunct to standard treatment modalities, i.e., CPAP, surgical interventions, and lifestyle modifications.

## 3. Literature Review

Obstructive sleep apnea (OSA) occurs as impaired breathing during sleeping. It predominantly affects adults, with a higher prevalence among males. In Poland, 3.4% of women and 11.2% of men report suffering from OSA [12]. In the US, growth from 34.3% to 46.2% is predicted by 2050 [13]. An episode of OSA appears as a temporary closure of the upper airways for at least 10 s which consequently causes a temporary hypoxia, hypercapnia, sympathetic nervous system activation, and increased blood pressure [14]. Untreated OSA affects daytime functioning due to chronic fatigue, which directly compromises reaction times to various stimuli. For example, OSA has been connected to an increased rate of traffic accidents among affected drivers [15].

The pathophysiology of OSA stems from a significant weakening of muscle tension, specifically the soft palate uvula, posterior pharyngeal wall, and tongue, which all collapse during sleep, thus causing obstruction of airways and disruption of ventilation [16]. Additional factors predisposing the airways to collapse are as follows: a reduced length of mandible, a posterior displacement of maxilla, a lower position of the hyoid bone, a hypertrophy of surrounding soft tissue, and narrowing of the pharyngeal space due to fat deposition in obesity [17]. The genetic diseases with craniofacial structural abnormalities like Crouzon’s, Apert’s, Pfeiffer‘s, and Pierre Robin’s syndromes also contribute to OSA [18]. It has been demonstrated that the shape of the tongue in individuals with OSA differs from that observed in patients without sleep apnea [19]. The case study conducted on 345 subjects proved that the anatomical structure of the upper airways in patients who suffer from OSA is narrower. OSA patients exhibited a longer and thicker tongue compared to non-OSA individuals [20].

In addition to the crucial local/anatomical predisposition, the general risk factors that enhance OSA frequency are age (elderly), sex, and obesity; however, the exact mechanism responsible for this phenomenon is not fully understood [20]. Obesity, particularly visceral obesity, is a major risk factor for the development of OSA, while central obesity is linked to a reduced space for the lung, thus loss of tension in the upper airways in a caudal direction and subsequent susceptibility of the pharynx to collapse [21]. Recent epidemiological data indicate that the global prevalence of obstructive sleep apnea (OSA) among older adults is approximately 36%. A systematic review and meta-analysis including 39 studies and over 33,000 participants reported a pooled prevalence of 35.9%. Despite considerable heterogeneity among the analyzed studies, the results consistently demonstrate that OSA represents a highly prevalent condition in the elderly population worldwide [22]. Additionally, a subgroup analysis considering age (<50 years vs. ≥50 years) demonstrated that the association between body mass index (BMI) and the severity of obstructive sleep apnea (OSA) was stronger among younger individuals, suggesting that adiposity exerts a greater influence on OSA pathophysiology in this age group [23]. The authors emphasized that possible mechanisms of increased frequency of OSA in the elderly were directly related to fat deposits at the parapharyngeal region and were responsible for elongation of the soft palate and the structural changes surrounding the pharynx [24]. A still unanswered but intriguing question remains: Why does OSA occur more frequently in men than women? According to Lam & Lam, this may be related to the anatomical and functional specificity of the upper airways’ anatomy, as well as the ventilatory response to arousals from sleep. Also, radiological studies proved that men accumulate more fat deposits around the pharynx [25]. It is believed that familial predisposition and genetic factors also play a role in the development of OSA. In the first-degree relatives, the risk of OSA episodes is 1.5 to 2-fold higher and increases with the number of affected relatives [26].

The confirmed behavioral factors that predispose to OSA are smoking and alcohol consumption [27]. Smoking is associated with a higher prevalence of snoring and sleep-related breathing disorders. Alcohol consumption extends the span of apneas, acting as a relaxant, which reduces the activity of the genioglossal muscle. Alcohol also diminishes awakenings, increases the frequency of airway closure episodes, and accelerates the severity of hypoxemia [28].

The OSA-related health risks result from the periodic drops in blood saturation and subsequent hypoxia of organs and tissues. To sustain homeostasis, both heart rate and blood pressure increase. The prolonged hypoxia during sleep results in the chronic elevation of blood pressure during the day. Consequently, this may promote the origin of both chronic hypertension and/or other cardiovascular disorders. It has been noted that individuals suffering from obstructive sleep apnea are at a greater risk of death from myocardial infarction or heart attack [20]. Other data showed that OSA is associated with resistance to insulin, provoking type 2 diabetes [29]. One of the potential, most severe consequences of OSA is brain hypoxia-related stroke. In addition to stroke, brain hypoxia during sleep impairs cognitive functions, resulting in problems with concentration, memory, and learning [30]. Consequently, long-term, untreated OSA can contribute to depression, anxiety, and other mental health problems. A scrutinized interview confirmed that loud snoring and frequent breathing interruptions impact patients’ partners’ sleep quality [31].

All of the methods listed in Table 2 yield beneficial results in the treatment of sleep apnea; however, due to low therapy comfort, they are not always accepted by patients who choose a dental prosthetic option, such as an MAD, for the elimination of nocturnal breathing disorders. An MAD is minimally invasive, thus offering higher user comfort.

### 3.1. Diagnosis of Obstructive Sleep Apnea

The diagnosis of obstructive sleep apnea includes both subjective and objective assessments. The subjective one primarily implicates snoring during sleep and daytime sleepiness. Additionally, important parameters that help establish a correct diagnosis include the identification of the risk factors in the patient. One of the fundamental diagnostic methods involves questionnaires completed by the patient. Confirmation of the presence and ongoing monitoring of these symptoms can be achieved using various surveys, such as the Epworth Sleepiness Scale (ESS) [33]. Another method for screening for OSA is the STOP-BANG questionnaire shown in Table 3 [34]. During this screening assessment, the patient completes a questionnaire and answers questions in a “Yes” or “No” format.

In addition to questionnaires, there is an objective method for monitoring and diagnosing sleep apnea and other sleep-related breathing disorders called polysomnography (PSG). PSG is a comprehensive recording of the physiological changes that occur during sleep. The examination is usually conducted in a sleep laboratory or at the patient’s home using a portable device. PSG typically involves the use of four to eight channels to measure the following: airflow through the nose, chest effort, electrocardiogram (ECG), pulse oximetry, electroencephalography (EEG), electrooculography (EOG), electromyography (EMG), sleeping position, and leg movements [36]. This overnight examination involves continuous monitoring of the patient’s condition using a set of sensors, including a pulse oximeter, an electrocardiogram (ECG), sensors detecting temperature, and those detecting pressure changes caused by exhaled air (to measure airflow through the nose and mouth). The nasal pressure transducer detects pressure changes during inspiration and expiration to monitor airflow and is used to identify hypopneas (30% reduction in airflow) [37]. Additionally, a microphone records snoring, while an electroencephalogram (EEG) and electrooculogram (EOG) assess whether the patient is asleep and define in which phase of sleep he is. Additionally, the examination includes limb movement monitoring. Altogether, the data collected during the PSG analysis allow for a conclusive diagnosis and help to define the severity of the disease. The results might be expressed by a number of respiratory incidents per hour of sleep, the apnea–hypopnea index (AHI), or the respiratory disturbance index (RDI). The PSG-based diagnosis must be reinforced with the RDI results, which happens in the case of 15 or more incidences of respiratory disturbances per hour, or 5 or more incidences per hour in concert with the presence of at least one of the following symptoms: fatigue, insomnia, awakening with a sensation of breathlessness, choking or gasping, involuntary sleep episodes during activities, witnessed apneic episodes, or loud snoring. The PSG results classify OSA severity into three categories—Mild OSA (RDI ≥ 5 and <15), Moderate (RDI ≥ 15 and ≤30), and Severe (RDI > 30) [38].

**Figure 2:**
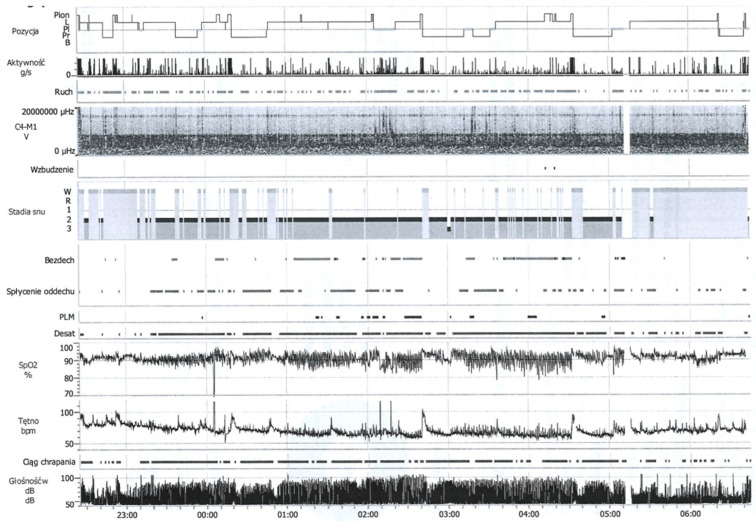
Results of a polysomnography test conducted in the Clinic of Otolaryngology, University Clinical Centre, Gdansk.

The main outcome of the polysomnography (PSG) study (Figure 2) is the apnea–hypopnea index (AHI), which reflects the severity of the obstructive sleep apnea. The AHI corresponds to the average number of apneas (complete cessation of airflow) and hypopneas (partial cessation of airflow) per hour of sleep. Based on AHI records, the severity of OSA is assessed (Table 4).

### 3.2. Treatment

After a scrutinized diagnosis, the patient should be informed about the pathophysiology of OSA, the risk factors, health consequences, and the available treatment options. This helps to understand the reason and potential consequences of OSA. Patients with obesity should be encouraged to reduce their body weight. Alcohol, smoking, and sedative drugs, which lower muscle tone, should be avoided as well. This initial education supports the subsequent treatment process.

The first-choice treatment plan for the moderate, severe, and mild OSA with coexisting symptoms is continuous positive airway pressure (CPAP) therapy. CPAP is delivered by a nasal mask connected to a device generating positive airway pressure. This prevents an airway collapse and improves breathing parameters.

Oral appliances represent a therapeutic option for a specific group of patients with obstructive sleep apnea. According to Nanda et al. (2025) [4], the primary recipients of this option are individuals with snoring or mild OSA who do not respond to behavioral methods or are not suitable candidates for the standard treatment. Nanda et al. (2025) [4] stated that it can also be used in patients with moderate or severe OSA who do not tolerate or refuse CPAP therapy. The third group comprises patients who refuse surgical intervention or have general medical contraindications to the procedure [4].

A paper titled “The Occlusal Side Effects of Mandibular Advancement Device Therapy in Adult Sleep Apnea Patients: A Systematic Review” has demonstrated that mandibular advancement using an oral appliance (OA) yields excellent therapeutic effects in the treatment of OSA without undesirable side effects [5]. In the study described by Kim, patients treated with an OA presented a significant improvement in oxygen saturation when snoring, while daytime sleepiness subsided instantly [6]. A different study showed that mandibular advancement contributed to airway patency [7]. A well-documented example of an MAD is the Thornton Adjustable Positioner (TAP), widely used for treating snoring and sleep apnea [8]. According to Lo Giudice, it keeps the mandible in a forward position during sleep, ensuring airway patency. TAP appliances are comfortable, do not cause mucosal irritation, and contain a titanium mechanism that allows for adjustable mandibular advancement [9]. Another device is the OrthoApnea NOA, produced by OrthoApnea dental lab in Malaga, Spain, which consists of two splints. Contrarily to TAP, the OrthoApnea splints are joined by a screw that adjusts mandibular position by increasing muscle tension in the upper airway. Neither TAP nor OrthoApnea requires additional tubes or masks. Studies conducted by Tan et al. (2025) proved that advancing the mandible by 8 mm using a splint significantly improves the success of obstructive sleep apnea treatment by increasing the volume of the upper airway [8].

Despite the benefits, researchers like Uyaner et al. (2024) emphasize the contraindications to MADs, such as temporomandibular joint disorders, severe periodontitis, and extensive partial edentulism [10]. Potential side effects include excessive saliva production or dry mucosa, transient tooth pain, discomfort due to muscle stiffness, and soreness. Additionally, the side effects of treating OSA might extend to cases of malocclusion. The effectiveness of oral advancement splint therapy for obstructive sleep apnea depends on the patient having healthy dentition, as the device is directly attached to the teeth. The forces exerted by the splint during use can potentially lead to changes in bite alignment [10]. The most common change observed during oral splint therapy is a reduction in overjet, which refers to the horizontal distance between the upper and lower incisors when the teeth are in occlusion. Additionally, patients may experience a decrease in the number of contact points between the molars. These changes usually appear early during treatment and tend to persist, with a slow progression over time [9]. The initial bite pattern has a significant impact on the extent of bite modifications after oral appliance therapy. Patients with a deep bite, characterized by a significant vertical overlap of the incisors, exhibit a certain degree of protection against overjet changes [10]. The risk of bite changes is also influenced by the design of the oral splint itself. A study by Uniken et al. (2020) suggests that splints primarily attached to the front teeth lead to faster and more pronounced bite alterations compared to those that cover the entire dentition [11].

A novel treatment for mild OSA available in dental offices is laser therapy with an Er:YAG laser. This method relies on heating tissues of the tongue base, uvula, soft palate, and palatal arches, which results in increased tissue elasticity with subsequent reduction in its vibration during breathing [39]. The treatment is performed three times at two-week intervals. One treatment lasts 15–20 min [40].

## 4. Conclusions

Obstructive sleep apnea (OSA) remains a significant and underdiagnosed public health issue with wide-reaching systemic consequences. Dentists are integral to early recognition, referral, and management, especially as dental sleep medicine evolves. Mandibular advancement devices (MADs) offer a patient-friendly, effective alternative for many individuals intolerant to CPAP, showing substantial reductions in AHI, improvement in oxygen saturation, and relief of daytime symptoms. Nevertheless, clinicians should remain vigilant for occlusal and dental side effects, tailoring long-term care and device selection to individual risk profiles. The potential of adjunct approaches, such as Er:YAG laser therapy, and the emerging evidence linking dental status to OSA risk, highlight the need for ongoing research and multidisciplinary coordination.

Ultimately, optimal OSA care demands seamless collaboration between dentists, sleep specialists, and other healthcare providers. Routine screening and integration of dental sleep medicine into practice should become standard for at-risk populations. Future studies are required to refine patient selection criteria, prevent treatment-related complications, and assess the long-term stability and efficacy of innovative therapies. By staying at the forefront of evidence-based care, dentistry can further enhance outcomes and quality of life for patients with sleep-disordered breathing.

## Figures and Tables

**Table 1:** Selected articles included in the systematic review.

Number of the Citation in the Publication	Article
[4]	Nanda N, Robertson A, Upton DC. “Patient Selection for Efficacious Mandibular Advancement Device Therapy in OSA.” Ann Otol Rhinol LaryngdES2025.
[5]	Rana A, Raut A, Mathur A. “The Occlusal Side Effects of Mandibular Advancement Device Therapy.” Cureus. 2023.
[6]	Kim HK, Kim ME. “Treatment Outcomes of Mandibular Advancement Devices in OSA.” J Oral Med Pain. 2023.
[7]	“Definition of an Effective Oral Appliance for Treatment of OSA and Snoring.” JDSM. 2025.
[8]	Tan WMN, Lye KW, Saffari SE, Poon CY. “MAD effectiveness in young OSA patients.” Sleep Sci Pract. 2025.
[9]	“Diagnostic & Therapeutic Indications of Different MAD Types for OSA.” 2025.
[10]	Uyaner A et al. “Impact of MADs on Occlusal Dynamics & Tooth Alignment.” Dent J. 2024.
[11]	Uniken Venema JAM et al. “Dental side effects of long-term OSA therapy.” Clin Oral Investig. 2020.

**Table 2:** Methods of treating obstructive sleep apnea.

Methods of Treating Obstructive Sleep Apnea [32]	
CPAP (Continuous Positive Airway Pressure)	This involves maintaining continuous positive airway pressure during sleep through the use of a nasal mask.
Lifestyle modifications and weight loss	This is particularly recommended for patients who are overweight or obese.
Positional therapy	This involves encouraging patients to sleep in specific positions to reduce the frequency of apneic episodes.
Hypoglossal Nerve Stimulation (HNS)	This therapy involves stimulating the hypoglossal nerve to help keep the airways open during sleep.

**Table 3:** STOP-BANG questionnaire [35].

STOP-BANG Questionnaire	YES	NO
Snoring: Do you snore loudly (enough to be heard through closed doors)?		
Fatigue: Do you often feel tired, exhausted, or sleepy during the day (e.g., falling asleep while driving)?		
Observations by others: Has anyone noticed that you stop breathing during sleep or choke/gasp?		
Blood pressure: Do you have hypertension or take medications for hypertension?		
BMI: Is your body mass index (BMI) greater than 35 kg/m^2^?		
Age: Are you over 50 years old?		
Neck circumference: Do you have a collar size of 41 cm or larger (for women) or 43 cm or larger (for men)?		
Gender: Are you male?		
For the general population, the results of the STOP-BANG questionnaire are as follows: **Low Risk of OSA**: “Yes” to 0–2 questions **Medium Risk of OSA**: “Yes” to 3–4 questions **High Risk of OSA**: “Yes” to 5–8 questions		

**Table 4:** OSA classification related to apnea–hypopnea index.

The Severity of Obstructive Sleep Apnea	Level of AHI
Mild OSA	5 to 15
Moderate OSA	15 to 30
Severe OSA	Above 30
**The Criteria for Children**	**Level of AHI**
Mild OSA	1 to 5
Moderate OSA	5 to 10
Severe OSA	Above 10

## Data Availability

The original data presented in the study are openly available in PubMed at https://pubmed.ncbi.nlm.nih.gov/.

## Supplementary Materials

The following supporting information can be downloaded at: https://www.mdpi.com/article/10.3390/dj14010062/s1, File S1: PRISMA 2020 Checklist [41].
